# Tuning the Inter-Chromophore Electronic Coupling in Perylene Diimide Dimers with Rigid Covalent Linkers

**DOI:** 10.3390/molecules30122513

**Published:** 2025-06-08

**Authors:** Guo Yu, Yixuan Gao, Yonghang Li, Yiran Tian, Xiaoyu Zhang, Yandong Han, Jinsheng Song, Wensheng Yang, Xiaonan Ma

**Affiliations:** 1 Institute of Molecular Plus, Tianjin University, Tianjin 300072, China; yuguo@tju.edu.cn (G.Y.); imp2019_gilvon@tju.edu.cn (Y.G.); lyh2450311523_@tju.edu.cn (Y.L.); yrtian08@tju.edu.cn (Y.T.); 2 Engineering Research Center for Nanomaterials, Henan University, Kaifeng 475004, China; xiaoyuz@henu.edu.cn (X.Z.); yandonghan@henu.edu.cn (Y.H.); songjs@henu.edu.cn (J.S.)

**Keywords:** perylene diimide, covalent dimer, electronic coupling, structural relaxation, excited state, molecular designing

## Abstract

The organic multi-chromophore system has been increasingly attractive due to the potential optoelectronic applications. The inter-chromophore electronic coupling (EC), i.e., *J*_Coul_ and *J*_CT_, plays a critical role in determining the relaxation path of the excited state. However, the molecular designing strategy for effective tuning of inter-chromophore EC is still challenging. In this computational work, we designed a series of perylene diimides (PDI) covalent dimers with rigid linking cores containing thiophene (Th) or phenyl (Ph) fragments and performed corresponding theoretical investigation to analyze the inter-PDI electronic coupling. Vibrational analysis indicated that the minimized excited state structural relaxation (ES-SR) can ensure the rigid inter-PDI geometry pre-defined by the topological characteristic of linking cores, leading to comparable |*J*_Coul_| on S_0_ and S_1_ states. The saddle-shaped linking cores allow collaborative tuning of inter-PDI dihedral (*α*) and slipping (*θ*) angles, leading to effective tuning of inter-PDI |*J*_Coul_| = 0–1000 cm^−1^. Our work provides a new molecular designing strategy for effective tuning of inter-chromophore EC for organic chromophores. By using a rigid inter-chromophore structure, the ignorable ES-SR allows simplified molecular designing without considering the plausible geometric difference between S_1_ and S_0_ states, which might be useful for future applications in organic optoelectronics.

## 1. Introduction

Inter-chromophore electronic coupling (EC) plays a key role in determining the excited state behavior of multi-chromophore systems and optoelectronic performance of organic semiconductors with ordered molecular packing [1,2,3,4,5,6,7]. In the weak coupling regime, photoinduced dynamics of chromophores can be dominated by the locally excited (LE) state. With enhanced inter-chromophore EC combining with further condition such as energy matching, the initially populated LE state may be obtained through charge transfer (CT) [8,9,10,11,12], symmetry-breaking charge separation (SB-CS) [13,14,15,16,17,18], singlet fission (SF) [19,20,21,22,23,24], and even formation of excimer/exciplex state [25,26,27,28,29,30,31]. Recently, photo-induced SB-CS [32,33,34] and SF [35,36,37] in covalent oligomers received great attention due to potential application in improving power conversion efficiency (PCE) of organic photovoltaics (OPV) [38,39,40,41,42,43]. For enabling efficient SB-CS and SF in organic multi-chromophore systems, a key task would be smart molecular designing to achieve delicate inter-chromophore EC [20,23,44], which remains challenging.

The inter-chromophore EC has been intensively discussed in the framework of the exciton theory pioneered by Davydov [45], Kasha [46,47,48], and Spano [49,50,51,52]. Briefly, inter-chromophore EC (*J*) can be described as the sum of Columbic coupling (*J*_Coul_) and CT-mediated super-exchange (*J*_CT_), i.e., *J* = *J*_Coul_ + *J*_CT_, [4,51,52] in which *J*_Coul_ and *J*_CT_ correspond to the long-range dipole-dipole interaction and frontier orbital overlapping in short-range, respectively. In particular, *J*_Coul_ can be tunable by molecular designing as it is associated with inter-chromophore geometry, i.e., inter-chromophore distance (*R*), dihedral angle (*α*), and slipping angle (*θ*), which has been described by [45,48,53,54].

(1) $$J_{Coul}=\frac{1}{4\pi \varepsilon_{0}}\frac{\mu^{2}\left(\cos\alpha -3\cos^{2}\theta \right)}{R^{3}}$$

The definition of *α* and *θ* angles can be seen in the illustration of Figure S1. In comparison, *J*_CT_ is a short-range interaction originating from frontier orbital overlapping and associated with electron (*t*_e_) and hole (*t*_h_) transfer integral, as well as adiabatic energy of CT (*E*_CT_) and the lowest-lying state (*E*_S1_) [51,55,56]. Please note that *J*_CT_ can also be greatly affected by geometric parameters, such as inter-chromophore slipping in a co-facial dimer [51], but it relies on a short inter-chromophore distance (typically a few angstroms).

(2) $$J_{CT}=-2\frac{t_{e}t_{h}}{E_{CT}-E_{S_{1}}}$$

Unlike excimer/exciplex formation, which relies on strong EC [30,57,58], efficient SB-CS and SF require moderate EC, corresponding to the “degraded” structure rather than fully stacked inter-chromophore geometry, i.e., inter-chromophore geometry should be deviated from the co-facial stacking geometry to reduce EC [59,60], which is often accompanied by changes of inter-chromophore dihedral (*α*) and slip angles (*θ*). Therefore, enormous efforts in molecular designing have been made to achieve the “degraded” inter-chromophore geometry. For instance, taking advantage of excited state structural relaxation (S_1_/S_0_ ES-SR), multi-chromophore systems with flexible linkers have been widely employed for facilitating efficient SB-CS and SF [59,60,61,62,63,64]. However, considerable S_1_/S_0_ ES-SR also leads to unpredictable EC in the sense of molecular designing. Recently, we demonstrated a new molecular designing strategy for this issue [65]. By using a saddle-shaped cyclooctatetrathiophene (COTh) core and fused linking with perylene diimides (PDI), optimized inter-PDI EC (|*J*_Coul_| ≈ 700 cm^−1^) was achieved, by which efficient inter-PDI SF (τ_SF_ = ~150 ps and ~150% triplet yield) was facilitated. Considering the rigid COTh structure and fused linking with PDIs, the inter-PDI geometry that can greatly affect *J*_Coul_ can be defined by the topological characteristics of COTh. In this sense, we should be able to achieve fine-tuning of inter-PDI EC (*J*_Coul_) only by modifying the structure of the rigid linking core, by which molecular designing of multi-chromophore systems can be simplified, i.e., only the geometry of the rigid core should be considered to facilitate the expected EC.

In this work, we further extended our molecular designing strategy for multi-chromophore systems. By using different rigid linkers with thiophene (Th) or phenyl (Ph) fragments, a series of PDI dimers was theoretically designed, leading to a broad tuning range of inter-PDI EC (0 < |*J*_Coul_| < 1000 cm^−1^). The vibrational analysis indicated that the S_1_/S_0_ ES-SR of rigid linking cores can be further suppressed by linking with PDI units, leading to comparable |*J*_Coul_| on S_0_ and S_1_ states, for which inter-PDI geometry was pre-defined by rigid linking cores. Considering the saddle-shaped feature of involved linking cores, our strategy enabled collaborative tuning of inter-PDI dihedral (*α*) and slipping (*θ*) angles, which can greatly neutralize the extension of inter-PDI distance (*R*) and lead to effective tuning of inter-PDI |*J*_Coul_|. Our work provides a new molecular designing strategy for fine-tuning of inter-chromophore EC for organic chromophores like PDI without disturbing S_1_/S_0_ ES-SR, which might be useful for future applications in organic optoelectronics.

## 2. Results and Discussion

### 2.1. Molecular Designing

**Figure 1:**
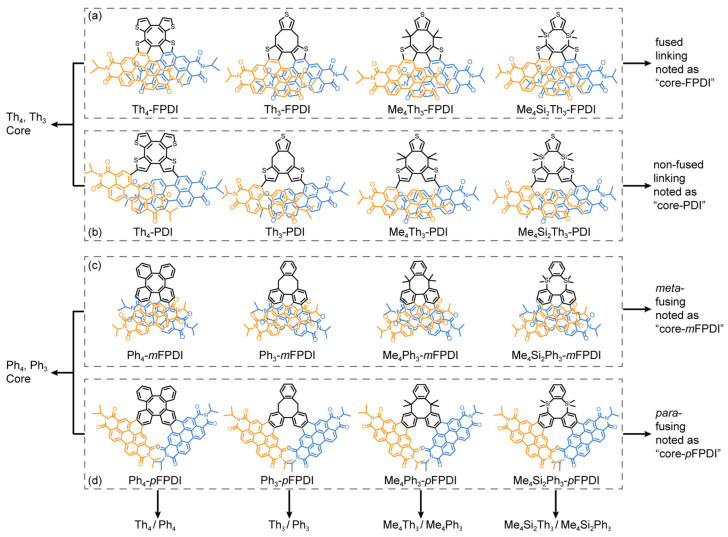
Illustrated chemical structures of investigated PDI dimers: (**a**) PDI dimers with Th-based core and fused linking, (**b**) PDI dimers with Th-based core and non-fused linking, (**c**) PDI dimers with Ph-based core and *meta*-fused linking, (**d**) PDI dimers with Ph-based core and *para*-fused linking.

In the present work, two structural features were considered for molecular designing of PDI dimers: (1) structure of rigid linking core; (2) linking manner between rigid core and PDI units. As illustrated in Figure 1a, we first modified the COTh (noted as Th_4_ in this work) core to the Th_3_ core, in which the structural symmetry can be reduced. The Me_4_Th_3_ and Me_4_Si_2_Th_3_ cores were subsequently designed to further enhance the structural rigidity. Two PDI units can be further introduced on each Th-based core with fused (rigid, Figure 1a) and non-fused (flexible, Figure 1b) linking, respectively. In comparison between PDI dimers illustrated in Figure 1a,b, we can further verify the key role of pre-defining inter-PDI geometry by rigid linking cores. Similarly, we replaced all Th units on linking the core with phenyl units, leading to corresponding cores of Ph_4_, Ph_3_, Me_4_Ph_3_, and Me_4_Si_2_Ph_3_. For each Ph-based core, PDI units can be fused to the linking core on *meta*- (Figure 1c) and *para*-position (Figure 1d) of the center cyclooctatetraene, leading to PDI dimers with variable EC due to changed inter-PDI distance (*R*), dihedral (*α*), and slipping angle (*θ*).

The optimized geometry on S_0_ and S_1_ states of the involved linking cores was calculated by using DFT and TDDFT approaches on B3LYP/6-311g** level, respectively. We employed the twisting dihedral angle (δ, as illustrated in Figure S2) for describing the saddle-shaped geometric feature of linking cores. As summarized in Table 1, all investigated Th- and Ph-based cores exhibited greatly reduced δ_S1_ than δ_S0_, corresponding to pronounced S_1_/S_0_ ES-SR. However, we found that S_1_/S_0_ ES-SR of cores can be greatly suppressed by linking with PDI units, leading to comparably twisted (60 to 70°) δ_S1_ and δ_S0_ angles. The calculated root of the mean of squared displacement (RMSD_S1/S0_) between S_1_ and S_0_ state also indicated less pronounced S_1_/S_0_ ES-SR of linking cores in PDI dimers, which might be explained by two aspects of factor [66,67]. Firstly, the steric effect induced by PDI units may suppress S_1_/S_0_ ES-SR of linking cores, which can be verified by lower core RMSD_S1/S0_ in non-fused dimers (i.e., Th_3_-PDI, etc.) than in corresponding fused dimers (i.e., Th_3_-FPDI, etc.). Meanwhile, considering the higher S_0_→S_1_ energy gap of the investigated linking cores (>3 eV) than the PDI unit (<2.5 eV), the cores might not be greatly involved in the S_0_→S_1_ transition of the corresponding PDI dimers. The linking cores with minimized S_1_/S_0_ ES-SR in PDI dimers ensured that the inter-PDI geometry can be pre-defined by rigid cores for tuning the inter-PDI *J*_Coul_.

### 2.2. Electronic Coupling

As described above, the inter-PDI EC in PDI dimers includes contributions of *J*_Coul_ and *J*_CT_, in which *J*_Coul_ is highly dependent on the inter-PDI geometry, i.e., inter-PDI distance (*R*), dihedral angle (*α*), and slipping angle (*θ*). Meanwhile, *J*_CT_ is a short-range interaction that is dependent on overlapping and alignment of frontier orbitals. The term of (cos*α* − 3cos^2^*θ*) involved in Equation (1) of *J*_Coul_ can usually be defined as an angular factor (κ) which determines the positive and negative values of *J*_Coul_. In exciton theory, *J*_Coul_ < 0 and *J*_Coul_ > 0 imply the J- (head-to-tail) and H-type (side-by-side) interaction among chromophores.

In contrast, *J*_CT_ is a sort of short-range interaction associated with frontier orbital overlapping, which is considerably dependent on inter-PDI geometry, only with short inter-PDI distance (typically a few angstroms). The *J*_Coul_ (Equation (1)) of all designed PDI dimers with S_0_ and S_1_ geometries was calculated together with corresponding *J*_CT_ (Equation (2)), leading to values of κ_S0_, κ_S1_, *J*_Coul_^S0^, *J*_Coul_^S1^, and *J*_CT_ summarized in Table 2. The geometric parameters of PDI dimers (*α*, *θ*, and *R*) for calculating *J*_Coul_ on S_0_ and S_1_ states can be seen in Table S1. The corresponding parameters for calculating inter-PDI *J*_CT_ were obtained by using the Multiwfn program [68,69], and are listed in Table S2.

As shown in Figure S3a, calculated *J*_CT_ of PDI dimers exhibited a jump-like dependence on inter-PDI distance, i.e., *J*_CT_ > 400 cm^−1^ in the area of *R* = 6–9 Å and *J*_CT_ < 200 cm^−1^ in the area of *R* = 9–12 Å, which is consistent with the short-range nature of *J*_CT_. Meanwhile, dependence of *J*_CT_ on *α* and *θ* angles can be barely observed (Figure S3b,c), which is dramatically different from the case of slipping perylene dimers with 3.5 Å stacking distance [51]. Please note that the positive/negative sign of *J*_CT_ is highly dependent on the sign of *t*_h_ and *t*_e_. However, a sign of *t*_h_ and *t*_e_ is determined by the phase assignment of the frontier orbital under a specific symmetry operation [52]. To avoid redundant discussion, we treated *t*_h_ and *t*_e_ with the definition by Wasielewski et al. [3], in which *t*_h_ and *t*_e_ can be defined with a different sign as moving a hole to one direction corresponds to moving an electron to the reverse direction.

**Figure 2:**
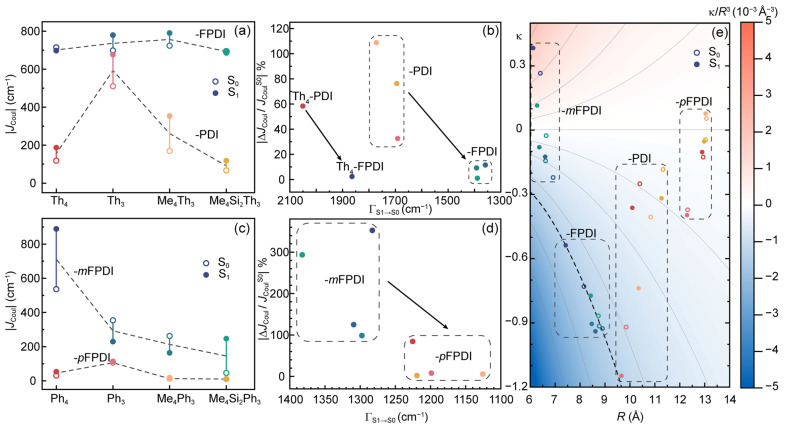
The calculated inter-PDI |*J*_Coul_| of Th- (**a**) and Ph-based (**b**) PDI dimers on S_0_ (circles) and S_1_ (dots) state. The relative change of *J*_Coul_ between S_1_ and S_0_ state was defined as |(*J*_Coul_^S1^ − *J*_Coul_^S0^)/*J*_Coul_^S0^|, which was plotted as a function of the total reorganization energy of S_1_→S_0_ transition for Th- (**c**) and Ph-based (**d**) PDI dimers. The contour map of κ/*R*^3^ depending on κ and *R* was plotted (**e**), on which data of Th- and Ph-based dimers were indicated as circles (S_0_ state) and dots (S_1_ state).

We further discuss the calculated inter-PDI *J*_Coul_ that is highly dependent on inter-PDI geometry in designed covalent dimers. As shown in Figure 2a, PDI dimers with fused linking to Th-based cores (Th-FPDIs) exhibited large *α* angle (60–70°) and slightly slipping (*θ* ≈ 50°) inter-PDI geometry on the S_1_ state, corresponding to a degraded stacking geometry with comparably strong inter-PDI EC (|*J*_Coul_| = 700–800 cm^−1^). In contrast, PDI dimers with flexible linking (Th-PDIs) exhibited greatly weakened EC (|*J*_Coul_| = 100–700 cm^−1^) than corresponding PDI dimers with fused linking. Compared to Th-FPDIs, inter-PDI geometry with non-fused linking can derive from the optimal geometry pre-defined by Th-based cores, leading to weakened EC. We further defined (*J*_Coul_^S1^ − *J*_Coul_^S0^)/*J*_Coul_^S0^ as an indicator for evaluating *J*_Coul_ changing between S_0_ and S_1_ geometry.

As shown in Figure 2b, PDI dimers with fused-linking exhibited minimized *J*_Coul_ changing (<10%), which is highly different from the Th-PDIs with up to 110% changing due to substantial S_1_/S_0_ ES-SR quantified by total reorganization energy of S_1_→S_0_ transition (Γ_S1→S0_). In the sense of rational molecular designing, Th-FPDIs with nearly identical *J*_Coul_ on S_0_ and S_1_ states are highly appreciated as S_1_/S_0_ ES-SR might be less considered. Regarding PDI dimers with Ph-based cores, it can be seen that *meta*-fusing resulted in stronger inter-PDI EC (|*J*_Coul_| up to 900 cm^−1^) than corresponding Ph-*p*FPDIs (|*J*_Coul_| = 10–100 cm^−1^, Figure 2c). Meanwhile, Ph-*m*FPDIs exhibited larger S_1_/S_0_ ES-SR (Γ_S1→S0_) than corresponding *para*-fused dimers, leading to S_1_/S_0_ changing of *J*_Coul_ up to 100% (Figure 2d). Thus, *para*-fused linking for Ph-based PDI dimers leads to weak inter-PDI EC, although it might be favored in the sense of rational molecular designing.

As described in Equation (1), angular factor (κ) and inter-PDI distance (*R*) play a central role in determining inter-PDI *J*_Coul_, for which we plotted a contour map of κ/*R*^3^ as a function of κ and *R*. As can be seen in Figure 2e, Th-FPDI, Th-PDI, Ph-*m*FPDI, and Ph-*p*FPDI dimers are located in different areas of the κ–*R* map, leading to their own feature on absolute values and S_1_/S_0_ changing of inter-PDI *J*_Coul_. The narrow κ (−0.6 to −0.9) and *R* (7–9 Å) distribution of Th-FPDI dimers resulted to considerable inter-PDI *J*_Coul_. Meanwhile, non-fused linking and substantial S_1_/S_0_ ES-SR of Th-PDI dimers lead to κ ranging from −0.2 to −1.2 with slightly enlarged inter-PDI *R* (9.5–11.5 Å), corresponding to greatly reduced *J*_Coul_ than Th-FPDI dimers.

On the other hand, the weakened |*J*_Coul_| of Ph-*p*FPDIs (*R* = 12–13 Å) in comparison with Ph-*m*FPDI dimers (*R* = 6–7 Å) can be attributed to the greatly enlarged inter-PDI *R* rather than changed κ. Meanwhile, it can be seen that each Ph-*p*FPDI dimer exhibited nearly identical κ and *R* on S_0_ and S_1_ states, indicating greatly suppressed S_1_/S_0_ ES-SR in comparison to Ph-*m*FPDI dimers. Our calculation on *J*_Coul_ of PDI dimers indicated that S_1_/S_0_ ES-SR might be a critical factor to be considered for rational molecular designing of multi-PDI systems, for which we further analyzed the origin of S_1_/S_0_ ES-SR in the sense of vibronic coupling effect in S_1_→S_0_ transition.

### 2.3. Vibrational Analysis

As discussed above, S_1_/S_0_ ES-SR is a critical factor for rational molecular designing of multi-PDI systems. In our recent works [31,70,71], we demonstrated that specific S_1_/S_0_ ES-SR motions can be associated with promoting vibrational modes, which can engage in S_1_→S_0_ electronic transition with high Huang-Rhys factor (*S*_k_) and reorganization energy contribution (λ_k_). For a multi-chromophore system, inter-chromophore *J*_Coul_ might also be sensitive to specific vibrational modes corresponding to collective motions that can vary inter-chromophore geometry [31,65]. To learn details on S_1_/S_0_ ES-SR of PDI dimers, we performed vibrational analysis on involved Th- and Ph-based cores, as well as corresponding PDI dimers. The Huang-Rhys factor (*S*_k_) and reorganization energy contribution (λ_k_) of each vibrational mode (*k*) engaged in S_1_→S_0_ electronic transition were calculated as *S*_k_ = (2ℏ)^−1^ω_k_Δ*Q*_k_^2^ and λ_k_ = 2^−1^ω_k_^2^Δ*Q*_k_^2^ by using the MOMAP program [72,73,74,75] based on electronic structure calculation. The mode displacement (Δ*Q*_k_) can be calculated with displacement components (Δ*D_i_*) along internal coordinate *i* as Δ*Q*_k_ = ∑*_i_*ζ_k*i*_Δ*D_i_*.

**Figure 3:**
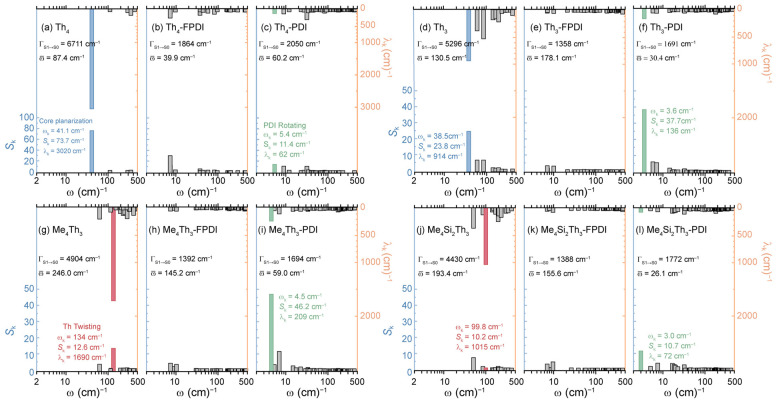
Vibrational analysis on Th-based linking cores (**a**,**d**,**g**,**j**), as well as corresponding PDI dimers with fused (**b**,**e**,**h**,**k**) and non-fused (**c**,**f**,**i**,**l**) linking. The Huang-Rhys factor (*S*_k_) and reorganization energy contribution (λ_k_) of vibrational modes engaged in S_1_→S_0_ transition were illustrated as a function of vibrational frequency (ω).

**Figure 4:**
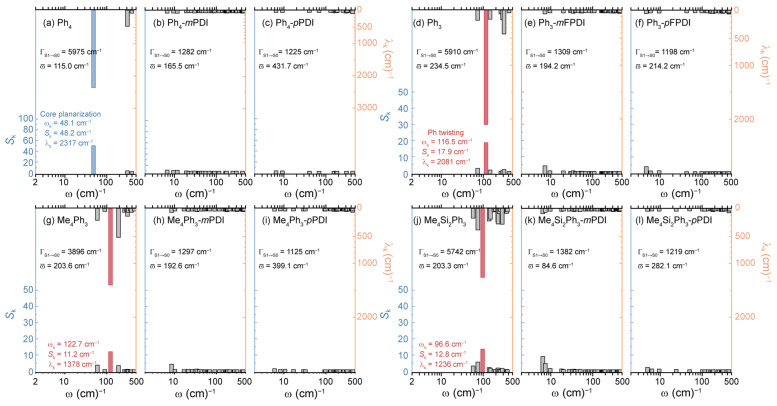
Vibrational analysis on Ph-based linking cores (**a**,**d**,**g**,**j**), as well as corresponding PDI dimers with *meta*-fused (**b**,**e**,**h**,**k**) and *para*-fused (**c**,**f**,**i**,**l**) linking. The Huang-Rhys factor (*S*_k_) and reorganization energy contribution (λ_k_) of vibrational modes engaged in S_1_→S_0_ transition were illustrated as a function of vibrational frequency (ω).

By summing the contribution of each mode, i.e., Γ_S1→S0_ = Σ_k_λ_k_, the total reorganization energy of the S_1_→S_0_ transition (Γ_S1→S0_) can be used for generalizing S_1_/S_0_ ES-SR of PDI dimers. As listed in Table 3, all Th- and Ph-based cores exhibited considerable Γ_S1→S0_ (up to 7000 cm^−1^). Meanwhile, the calculated Γ_S1→S0_ of corresponding PDI dimers was observed to be greatly reduced to 1000–2000 cm^−1^, indicating the suppressed λ_k_ of promoting modes associated with collective motion of PDI units, which is consistent with less pronounced S_1_/S_0_ ES-SR observed by RMSD_S1/S0_. Since the collective motion of PDI units is largely correlated with low-frequency modes of Th- and Ph-cores, we could resolve S_1_/S_0_ ES-SR affecting inter-PDI geometry by extracting corresponding modes of Th- and Ph-cores. The calculated results of vibrational analysis for Th- and Ph-cores can be seen in Figure 3 and Figure 4, together with corresponding PDI dimers.

As shown in Figure 3, Γ_S1→S0_ of Th_4_ core was dominated by a promoting mode (*S*_k_ = 73.7 cm^−1^, λ_k_ = 3020 cm^−1^) at ω_k_ = 14.1 cm^−1^ (noted as mode 1, blue colored), corresponding to planarized motion on S_1_ state (see Figure S4). In the S_1_→S_0_ transition of Th_3_, Me_4_Th_3_, and Me_4_Si_2_Th_3_ cores, mode 1 might be suppressed due to the structural tension. The Me_4_Th_3_ and Me_4_Si_2_Th_3_ cores exhibited an extra promoting mode with considerable λ_k_ contribution at ω_k_ = 100–140 cm^−1^ (noted as mode 2, red colored), corresponding to twisting motion of Th units (see Figure S4). The vibrational analysis on PDI dimers indicated that λ_k_ contribution of modes 1 and 2 was greatly reduced in the S_1_→S_0_ transition of all Th-based PDI dimers, implying suppressed S_1_/S_0_ ES-SR associated with modes 1 and 2, which ensures the inter-PDI geometry pre-defined by linking cores.

Intriguingly, we observed a new promoting mode located in ω_k_ < 10 cm^−1^ region (noted as mode 3, green colored) with considerable *S*_k_ and contribution to Γ_S1→S0_ of Th-PDI dimers. As can be seen in Figure S4, mode 3 of Th-PDI dimers exhibited the largest displacement toward the opposite direction in the ending sides of PDI units, as well as the minimized displacement in spatial center of PDI units, corresponding to a rotational motion of PDI units along the linking bond between PDI and Th cores. Such a rotational motion can be greatly limited by fused linking in Th-FPDIs, for which inter-PDI geometry can be pre-defined by Th cores. However, with single bond linking, the presence of mode 3 can lead to considerable S_1_/S_0_ ES-SR of Th-PDI dimers, resulting in greatly relaxed inter-PDI geometry with undermined |*J*_Coul_|.

We subsequently performed vibrational analysis on the S_1_→S_0_ transition of the Ph-based cores and corresponding PDI dimers. As shown in Figure 4, Ph-based cores exhibited very similar promoting modes of Th-based cores discussed above, i.e., mode 1 (ω_k_ = 48.1 cm^−1^) for Ph_4_ core and mode 2 (ω_k_ = 100–120 cm^−1^) for Th_3_, Me_4_Th_3_, and Me_4_Si_2_Th_3_ cores, corresponding to planarized and Ph twisting motions (see Figure S5), respectively. With the fused linking, Ph-*m*FPDI and Ph-*p*FPDI dimers exhibited an ignorable contribution of low-frequency modes to Γ_S1→S0_, indicating minimized S_1_/S_0_ ES-SR, which can ensure inter-PDI geometry pre-defined by linking cores. Please note that vibrational modes in the high-frequency regime (>500 cm^−1^) can also contribute to Γ_S1→S0_ as well. However, high-frequency modes usually correspond to localized motions without affecting inter-PDI geometry for *J*_Coul_, which can be ignored in the discussion of the present work.

### 2.4. Further Discussion

**Figure 5:**
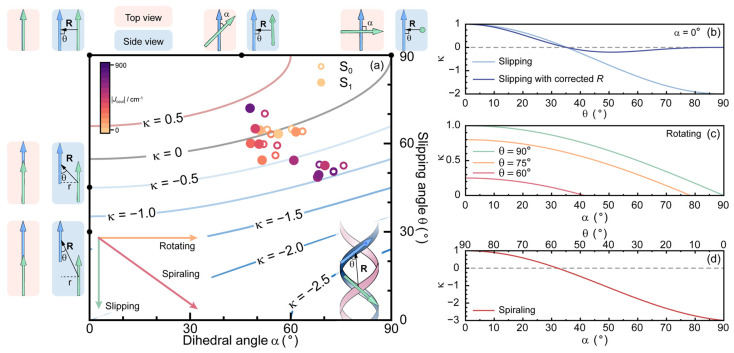
(**a**) Contour plotting of angular factor (κ) as a function of inter-PDI dihedral angle (*α*) and slipping angle (*θ*); S_0_ (circles) and S_1_ (dots) geometry of investigated PDI dimers were marked with colors scaling of |*J*_Coul_|. The evolution of κ in inter-PDI slipping and slipping with corrected *R* (**b**), rotating (**c**), and spiraling (**d**).

As mentioned above, inter-PDI EC plays a key role in determining the excited-state relaxation path of multi-chromophore PDI model systems, in which *J*_Coul_ is highly sensitive to inter-PDI geometry, i.e., angular factor κ and inter-PDI distance *R*. As shown in Figure 5a, we plotted a contour map of κ as a function of inter-PDI *α* and *θ* angles. The κ of PDI dimers investigated in this work ranges from −1.0 to 0.5, leading to |*J*_Coul_| tuning between 0 and 1000 cm^−1^, which is a considerable tuning range and may benefit from our strategy of molecular designing. By using rigid cores and fused covalent linking, the inter-PDI geometry of PDI dimers can be pre-defined by the topological characteristics of linking cores. As a result, collaborative modification of both inter-PDI *α* and *θ* angles can be achieved, leading to effective tuning of *J*_Coul_.

As shown in Figure 5d, collaborative changing of inter-PDI *α* (0°–90°) and θ (90°–0°) corresponds to a broad range of κ (1 to −3), which might lead to highly effective tuning of inter-PDI *J*_Coul_. For instance, inter-PDI *α* and *θ* in our designed PDI dimers can be changed in a relatively small range, i.e., 45°–75° and 75°–45°, respectively. However, collaborative changing of inter-PDI *α* and *θ* leads to a broad κ range (0.5 to −1.0), which is broader than the theoretically maximized tuning range by *α* (κ = 1 to 0) and *θ* (κ = 1 to −0.2) individually. As a result, a broad κ range by collaborative changing of inter-PDI *α* and *θ* can enable highly effective tuning of inter-PDI *J*_Coul_.

**Figure 6:**
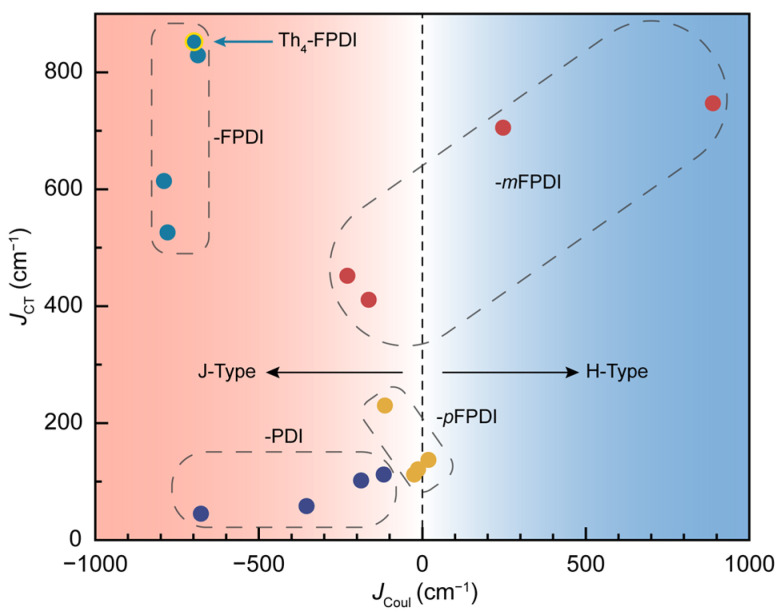
The correlated map of calculated *J*_Coul_ and *J*_CT_ for PDI dimers investigated in this work. The Th-FPDI, Th-PDI, Ph-*m*FPDI, and Ph-*p*FPDI dimers were marked by dashed boxes.

Last but not least, the plausible excited-state relaxation path of investigated PDI dimers can be discussed with the calculated *J*_Coul_ and *J*_CT_. As shown in Figure 6, Th-FPDI dimers mostly exhibited large |*J*_Coul_|, large |*J*_CT_|, and small |*J*_Coul_ + *J*_CT_|, which is consistent with the condition for plausible electronic state mixing described by Hariharan, Wasielewski, and co-workers [2,3,4,76]. In this type of PDI dimer, Th_4_-FPDI has been experimentally demonstrated to undergo SF to generate ^1^(TT) species by our ultrafast spectroscopic investigation [65], which can be regarded as the dephasing product of the mixed state. Furthermore, Ph-*p*FPDI dimers and most of Th-PDI dimers exhibited small |*J*_Coul_|, small |*J*_CT_|, and long inter-PDI distance, which are highly plausible to relax through incoherent SB-CS in a highly polar medium. For Ph_4_-*m*FPDI with huge |*J*_Coul_| (~900 cm^−1^) and close inter-PDI stacking, the excimer-like state can be expected through an incoherent relaxation.

## 3. Calculational Methods

All electronic structure calculations of designed cores and PDI-based dimers were performed using the Gaussian 16 software package [77]. The geometric structure of the investigated emitters was optimized on both ground (S_0_) and singlet (S_1_) excited states at B3LYP/6-311G** level, while no imaginary frequency was found by frequency analysis. The DFT and TDDFT optimized geometries of PDI-based dimers with root of the mean of squared displacement were analyzed and rendered by using the VMD 1.9.3 program [78]. The molecular orbitals analysis of the corresponding excited states was performed by using the Multiwfn program [68,69]. The Huang-Rhys (HR) factor (*S*_k_) and reorganization energy contribution (λ_k_) of each vibrational mode were calculated by using MOMAP 2021A on the basis of frequency analysis of corresponding states [72,73,74,75]. We further estimated the reorganization energy contribution (λ_k_) of each vibrational mode between the S_1_ and S_0_ states.

## 4. Conclusions

To summarize, we performed molecular designing of a series of PDI covalent dimers with rigid linking cores containing thiophene (Th) or phenyl (Ph) fragments and investigated the inter-PDI electronic coupling (*J*_Coul_ and *J*_CT_). The vibrational analysis on S_1_→S_0_ transition indicated that the S_1_/S_0_ ES-SR of rigid linking cores can be further suppressed by linking with PDI units, leading to comparable |*J*_Coul_| on S_0_ and S_1_ states, for which inter-PDI geometry can be pre-defined by rigid linking cores. Benefiting from the saddle-shaped feature of involved linking cores, our strategy enabled collaborative tuning of inter-PDI dihedral (*α*) and slipping (*θ*) angles, which can lead to effective tuning of inter-PDI |*J*_Coul_| = 0–1000 cm^−1^. Our work provides a new molecular designing strategy for fine-tuning of inter-chromophore EC for organic chromophores like PDI without disturbing S_1_/S_0_ ES-SR, which might be useful for future applications in organic optoelectronics.

## Figures and Tables

**Table 1:** The DFT/TDDFT calculated twisting angle (δ) of the investigated linking cores on S_0_/S_1_ states and corresponding RMSD_S1/S0_ with and without linking with PDI units.

Without PDI Units				With PDI Units			
	δ_S0_ (°)	δ_S1_ (°)	RMSD_S1/S0_ (Å)		δ_S0_ (°)	δ_S1_ (°)	RMSD_S1/S0_ (Å)
Th_4_	47.7	22.9	0.51	-FPDI	63.9	50.2	0.23
				-PDI	47.1	33.9	0.24
Th_3_	29.8	1.4	0.42	-FPDI	76.5	70.6	0.06
				-PDI	58.9	57.4	0.02
Me_4_Th_3_	64.6	39.1	0.19	-FPDI	78.0	72.6	0.06
				-PDI	64.6	62.7	0.03
Me_4_Si_2_Th_3_	60.7	30.5	0.26	-FPDI	76.6	71.4	0.07
				-PDI	57.7	57.2	0.04
Ph_4_	61.2	42.4	0.45	-*m*FPDI	59.8	57.4	0.05
				-*p*FPDI	59.8	57.7	0.04
Ph_3_	60.1	34.7	0.21	-*m*FPDI	65.5	63.1	0.04
				-*p*FPDI	46.6	43.7	0.05
Me_4_Ph_3_	62.0	43.2	0.17	-*m*FPDI	66.3	64.4	0.04
				-*p*FPDI	60.1	57.6	0.03
Me_4_Si_2_Ph_3_	62.4	35.2	0.23	-*m*FPDI	64.2	57.5	0.08
				-*p*FPDI	61.9	60.6	0.06

**Table 2:** Calculated angular factor (κ) and corresponding *J*_Coul_ of investigated PDI dimers on both S_0_ and S_1_ geometries, while *J*_CT_ of each PDI dimers was calculated as well.

	Noted as	κ_S0_	κ_S1_	*J*_Coul_^S0^ (cm^−1^)	*J*_Coul_^S1^ (cm^−1^)	*J*_CT_ (cm^−1^)
Th_4_-FPDI	Th-FPDIs	−0.73	–0.54	−714	−698	852
Th_3_-FPDI		–0.93	–0.94	−699	−779	526
Me_4_Th_3_-FPDI		–0.92	–0.90	−721	−790	614
Me_4_Si_2_Th_3_-FPDI		–0.87	–0.85	−691	−686	829
Th_4_-PDI	Th-PDIs	–0.25	–0.36	−118	−187	102
Th_3_-PDI		–0.92	–1.15	−511	−677	45
Me_4_Th_3_-PDI		–0.41	–0.74	−170	−354	58
Me_4_Si_2_Th_3_-PDI		–0.18	–0.32	−67	−118	112
Ph_4_-*m*FPDI	Ph-*m*FPDIs	0.27	0.38	536	889	747
Ph_3_-*m*FPDI		–0.22	–0.12	−354	−229	452
Me_4_Ph_3_-*m*FPDI		0.05	0.08	−263	−164	411
Me_4_Si_2_Ph_3_-*m*FPDI		–0.03	0.12	−47	247	705
Ph_4_-*p*FPDI	Ph-*p*FPDIs	0.18	–0.10	−31	−25	112
Ph_3_-*p*FPDI		–0.37	–0.40	−106	−114	230
Me_4_Ph_3_-*p*FPDI		–0.14	–0.08	13	19	137
Me_4_Si_2_Ph_3_-*p*FPDI		–0.04	–0.05	−11	−13	121

**Table 3:** Calculated total reorganization energy of S_1_→S_0_ transition (Γ_S1→S0_) for linking cores and corresponding PDI dimers.

	Γ_S1→S0_ (cm^−1^)		
	Th-based core	Th-FPDIs	Th-PDIs
Th_4_	6711	1864	2050
Th_3_	5296	1358	1691
Me_4_Th_3_	4904	1392	1694
Me_4_Si_2_Th_3_	4430	1388	1772
	Ph-based core	Ph-*m*FPDIs	Ph-*p*FPDIs
Ph_4_	5975	1282	1225
Ph_3_	5910	1309	1198
Me_4_Ph_3_	3896	1297	1125
Me_4_Si_2_Ph_3_	5742	1382	1219

## Data Availability

Data is contained within the article or Supplementary Materials.

## Supplementary Materials

The following supporting information can be downloaded at: https://www.mdpi.com/article/10.3390/molecules30122513/s1, Figure S1. Illustrated (a) dihedral angles (α), (b) slipping angles (θ) and distances (R) between PDIs; Figure S2. Illustrated twisting dihedral angle (δ) for describing the saddle-shaped geometric feature of linking cores; Figure S3. Calculated JCT changes with the (a) distance (R), (b) dihedral angle (α) and (c) slip angle (θ) between PDIs; Figure S4. Illustrated promoting modes of Th_4_(a), Th_3_(b), Me_4_Th_3_(c) and Me_4_Si_2_Th_3_(d); Figure S5. Illustrated promoting modes of Th_4_-PDI (a), Th_3_-PDI (b), Me_4_Th_3_-PDI (c) and Me_4_Si_2_Th_3_-PDI (d); Figure S6. Illustrated promoting modes of Ph_4_ (a), Ph_3_(b), Me_4_Ph_3_(c) and Me_4_Si_2_Ph_3_(d); Figure S7. The detailed evolution of angular factor (κ) in inter-PDI slipping with corrected *R*; Table S1. The geometric parameters used for calculating the *J_Coul_* of PDI dimers under the S0/S1 geometries optimized based on DFT/TDDFT; Table S2. The corresponding data for calculating the *J_CT_* of PDI dimers.
