# Supplementary material for: Tuning the Inter-Chromophore Electronic Coupling in Perylene Diimide Dimers with Rigid Covalent Linkers
Source: Molecules. 2025 Jun 8;30(12):2513. doi: 10.3390/molecules30122513 (PMC12195724; doi:10.3390/molecules30122513)
Supplement: Supplementary file 1 [file molecules-30-02513-s001.zip › molecules-3664220-supplementary.pdf]

Supplementary Materials

Tuning the Inter-Chromophore Electronic Coupling in Perylene Diimide Dimers with Rigid Covalent Linkers

Guo Yu [1], Yixuan Gao [1], Yonghang Li [1], Yiran Tian [1], Xiaoyu Zhang [2], Yandong Han [2], Jinsheng Song [2], Wensheng Yang [1,2,*] and Xiaonan Ma [1,*]

[1] Institute of Molecular Plus, Tianjin University, Tianjin 300072, China; yuguo@tju.edu.cn (G.Y.); imp2019_gilvon@tju.edu.cn (Y.G.); lyh2450311523_@tju.edu.cn (Y.L.); yrtian08@tju.edu.cn (Y.T.)
[2] Engineering Research Center for Nanomaterials, Henan University, Kaifeng 475004, China; xiaoyuz@henu.edu.cn (X.Z.); yandonghan@henu.edu.cn (Y.H.); songjs@henu.edu.cn (J.S.)
* Correspondence: wsyang@henu.edu.cn (W.Y.); xiaonanma@tju.edu.cn (X.M.)

Contents

Section	Content	Page
S1	Definition of dihedral and slipping angles	S3
	Figure S1	S3
S2	Saddle-shaped geometric feature of linking cores	S3
	Figure S2	S3
S3	The J_{Coul} and J_{CT} calculation of PDI dimers	S4
	Table S1	S4
	Table S2	S4
	Figure S3	S5
S4	Optimized geometry of designed linking core and PDI dimers	S6
	Figure S4	S6
	Figure S5	S6
	Figure S6	S7
S5	Detailed evolution of angular factor	S8
	Figure S7	S8

Section S1. Definition of dihedral and slipping angles

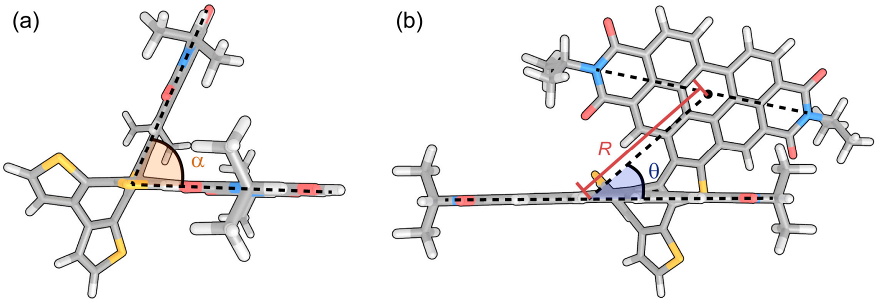

Figure S1. Illustrated (a) dihedral angles (α), (b) slipping angles (θ) and distances (*R*) between PDIs.

Section S2. Saddle-shaped geometric feature of linking cores

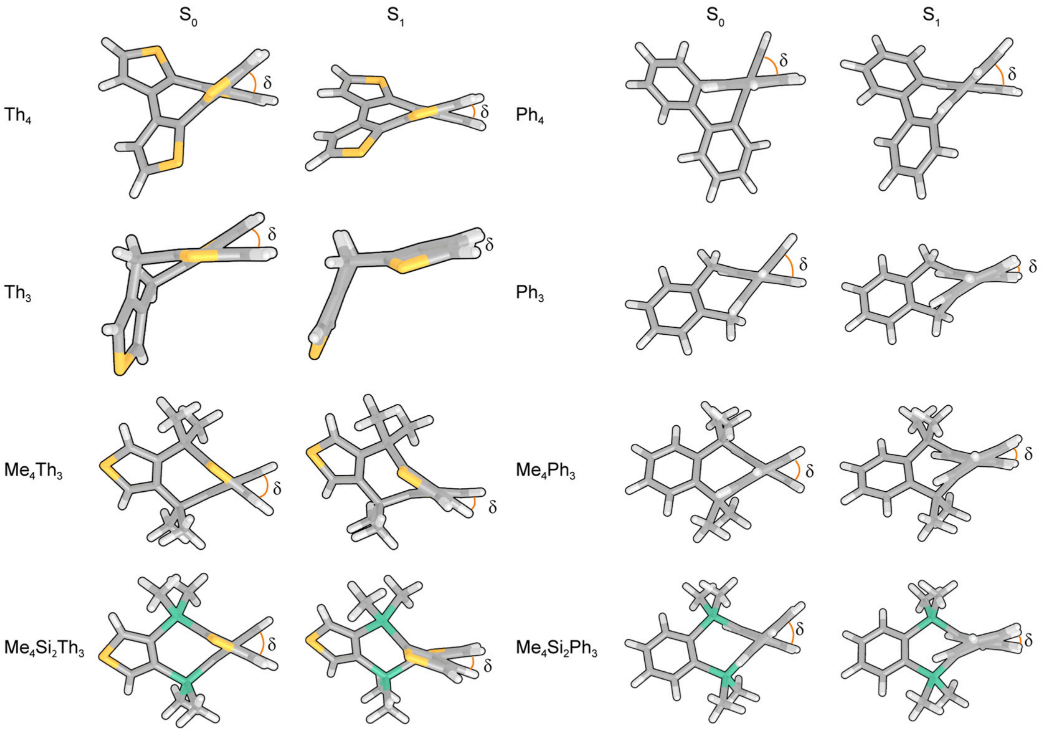

Figure S2. Illustrated twisting dihedral angle (δ) for describing the saddle-shaped geometric feature of linking cores.

Section S3. The J_{Coul} and J_{CT} calculation of PDI dimers

Table S1. The geometric parameters used for calculating the J_{Coul} of PDI dimers under the S_0/S_1 geometries optimized based on DFT/TDDFT.

	Noted as	J_{Coul}^{S0} (cm^{-1})			J_{Coul}^{S1} (cm^{-1})		
		α (°)	θ (°)	R (Å)	α (°)	θ (°)	R (Å)
Th_4-FPDI	Th-FPDIs	68.6	52.8	8.2	61.0	54.3	7.4
Th_3-FPDI		72.8	50.3	8.9	68.1	48.6	8.6
Me_4Th_3-FPDI		72.8	50.6	8.8	68.4	49.3	8.5
$Me_4Si_2Th_3$-FPDI		75.8	52.5	8.7	70.2	52.5	8.4
Th_4-PDI	Th-PDIs	83.6	69.7	10.4	88.3	68.8	10.1
Th_3-PDI		66.5	48.5	9.9	61.2	42.5	9.7
Me_4Th_3-PDI		78.3	63.2	10.8	61.7	50.5	10.4
$Me_4Si_2Th_3$-PDI		42.4	56.3	11.3	37.7	52.6	11.3
Ph_4-*m*FPDI	Ph-*m*FPDIs	52.3	70.2	6.4	47.9	72.0	6.1
Ph_3-*m*FPDI		56.0	59.3	6.9	50.6	59.8	6.6
Me_4Ph_3-*m*FPDI		51.9	59.8	6.6	48.0	60.0	6.4
$Me_4Si_2Ph_3$-*m*FPDI		54.1	63.2	6.6	49.4	65.0	6.3
Ph_4-*p*FPDI	Ph-*p*FPDIs	63.5	64.1	12.9	61.6	63.9	12.9
Ph_3-*p*FPDI		55.5	56.0	12.3	51.5	54.3	12.3
Me_4Ph_3-*p*FPDI		53.0	64.7	13.1	50.8	64.5	13.0
$Me_4Si_2Ph_3$-*p*FPDI		60.3	64.9	13.0	56.3	63.3	13.0

Table S2. The corresponding data for calculating the J_{CT} of PDI dimers.

	Noted as	t_h (cm^{-1})	t_e (cm^{-1})	E_{CT} (cm^{-1})	E_{S1} (cm^{-1})
Th_4-FPDI	Th-FPDIs	−757	11	20344	20324
Th_3-FPDI		−290	133	19869	19723
Me_4Th_3-FPDI		−295	117	19739	19626
$Me_4Si_2Th_3$-FPDI		−271	44	19665	19636
Th_4-PDI	Th-PDIs	−776	102	19181	17628
Th_3-PDI		−42	112	16647	16438
Me_4Th_3-PDI		−247	33	16639	16358
$Me_4Si_2Th_3$-PDI		−154	57	16786	16629
Ph_4-*m*FPDI	Ph-*m*FPDIs	−2121	413	21485	19138
Ph_3-*m*FPDI		−2387	267	22015	19198
Me_4Ph_3-*m*FPDI		−2252	298	20894	17634
$Me_4Si_2Ph_3$-*m*FPDI		−2292	390	21669	19137
Ph_4-*p*FPDI	Ph-*p*FPDIs	−129	81	19065	18879
Ph_3-*p*FPDI		−232	200	19008	18604
Me_4Ph_3-*p*FPDI		−207	61	18801	18616
$Me_4Si_2Ph_3$-*p*FPDI		−186	46	19117	18977

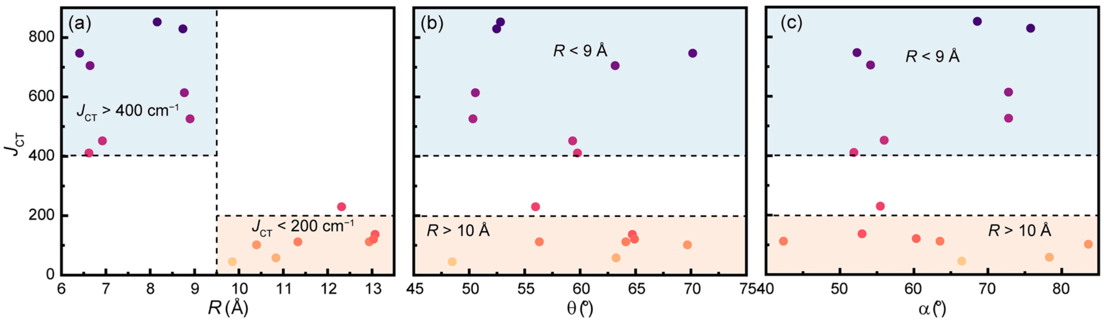

Figure S3. Calculated J_{CT} changes with the (a) distance (R), (b) dihedral angle (α) and (c) slip angle (θ) between PDIs.

Section S4. Optimized geometry of designed linking cores and PDI dimers

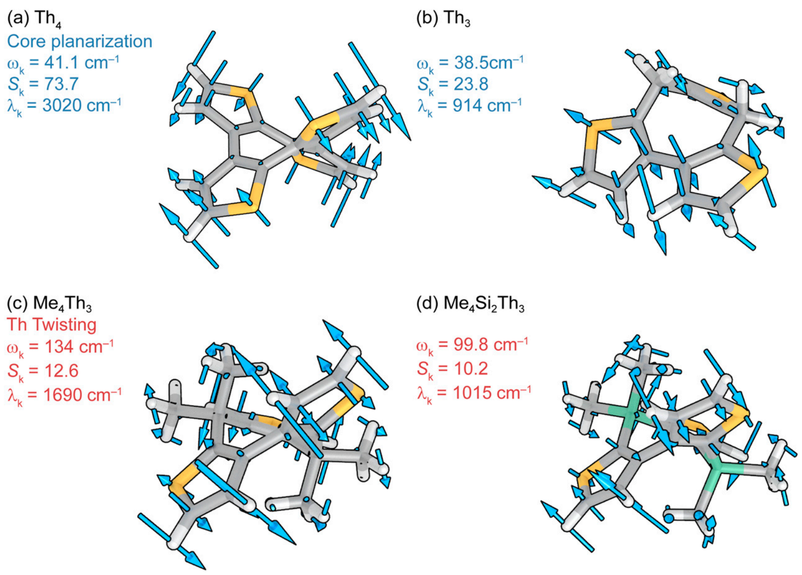

Figure S4. Illustrated promoting modes of Th_4 (a), Th_3(b), Me_4Th_3(c) and $Me_4Si_2Th_3$(d).

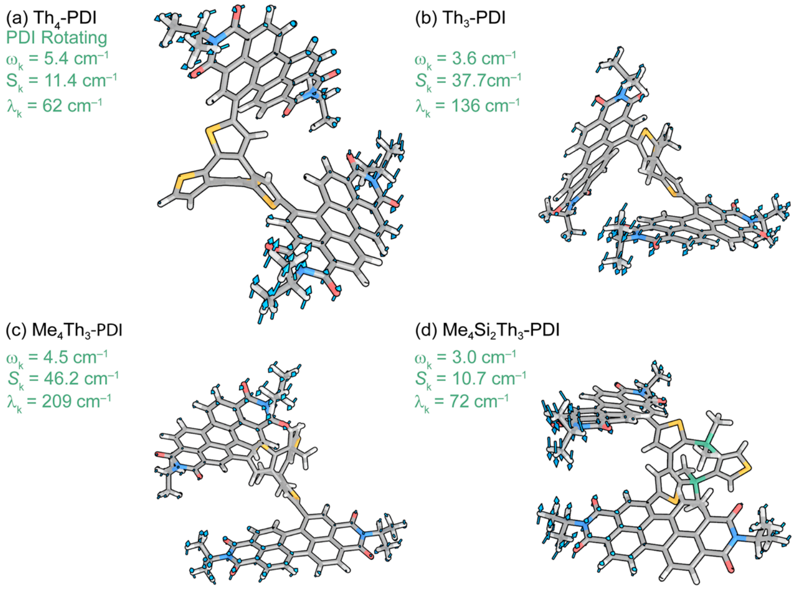

Figure S5. Illustrated promoting modes of Th_4-PDI (a), Th_3-PDI (b), Me_4Th_3-PDI (c) and $Me_4Si_2Th_3$-PDI (d).

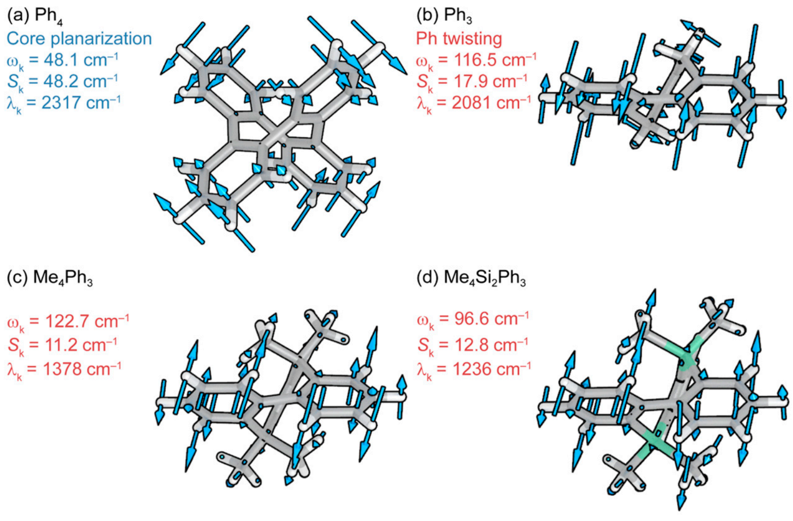

Figure S6. Illustrated promoting modes of Ph_4 (a), Ph_3(b), Me_4Ph_3(c) and $Me_4Si_2Ph_3$(d).

Section S5. Detailed evolution of angular factor

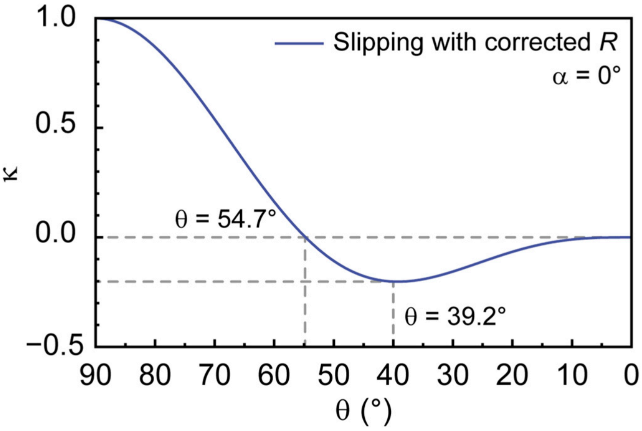

Figure S7. The detailed evolution of angular factor (κ) in inter-PDI slipping with corrected *R*.
